# Molecular Dynamics of Neutral Polymer Bonding Agent (NPBA) as Revealed by Solid-State NMR Spectroscopy

**DOI:** 10.3390/molecules19011353

**Published:** 2014-01-22

**Authors:** Wei Hu, Yongchao Su, Lei Zhou, Aimin Pang, Rulin Cai, Xingang Ma, Shenhui Li

**Affiliations:** 1 Hubei Institute of Aerospace Chemotechnology, Xiangyang 441003, China; 2 State Key Laboratory of Magnetic Resonance and Atomic and Molecular Physics, Wuhan Institute of Physics and Mathematics, the Chinese Academy of Sciences, Wuhan 430071, China

**Keywords:** bonding polymer, NPBA, structure, dynamics, solid-state NMR

## Abstract

Neutral polymer bonding agent (NPBA) is one of the most promising polymeric materials, widely used in nitrate ester plasticized polyether (NEPE) propellant as bonding agent. The structure and dynamics of NPBA under different conditions of temperatures and sample processing are comprehensively investigated by solid state NMR (SSNMR). The results indicate that both the main chain and side chain of NPBA are quite rigid below its glass transition temperature (T_g_). In contrast, above the T_g_, the main chain remains relatively immobilized, while the side chains become highly flexible, which presumably weakens the interaction between bonding agent and the binder or oxidant fillers and in turn destabilizes the high modulus layer formed around the oxidant fillers. In addition, no obvious variation is found for the microstructure of NPBA upon aging treatment or soaking with acetone. These experimental results provide useful insights for understanding the structural properties of NPBA and its interaction with other constituents of solid composite propellants under different processing and working conditions.

## 1. Introduction

Solid propellants of the composite type, a series of heterogeneous energetic materials composed of intimately mixed fuel, oxidizer and polymeric binder, have a wide range of applications in the chemical, chemical engineering, ordnance and propulsion industries and research communities. As an important component, the binding matrix formed between binding agents and curative agents serves as the interaction network and assures the mechanical properties of propellants. A good binding matrix can maintain the geometric integrity of the propellant ingredients even when subject to severe extreme temperature and pressure conditions. Poor performance of the binding matrix, e.g., debonding from the solid fillers, greatly compromises the efficiency of propellants. In order to improve the binding ability, a small amount of bonding agents is usually incorporated into propellants [1,2,3,4,5,6,7,8,9,10]. Ever since the 1960s, an array of bonding agents such as aziridine, alkanolamine, polyamine and their derivatives were developed for hydroxyl-terminated polybutyldiene (HTPB) propellants. To increase the interface bonding strength of polyether binders and nitrate fillers in nitrate ester plasticized polyether (NEPE) propellant, the neutral polymeric bonding agent (NPBA) was proposed by Kim *et al.* in 1990s [11,12]. It has repetitive aliphatic main chains and branched side chains of varied lengths, showing strong affinity to the polar filters. In addition, the hydroxyl-group rich side chains interact with the binder matrix, promoting the interfacial bonding strength, defined as stretching amplitue of the binding interface between propellants and linear parts [13,14].

Despite many studies characterizing the physical, chemical and mechanical properties of NPBA agents, the molecular properties, describing the structural and dynamic basis of their affinity to binding matrix, remains largely unknown. For example, the tensile strength of NEPE propellant with NPBA as bonding agent decreased significantly when the temperature increased from 298 K to 343 K [15]. Such an observation suggests that the glass transition of this polymeric molecule resulting from the temperature change modulates its binding function. Thus, it becomes important to investigate the dependence of the structure and dynamics on temperature changes, which can provide a mechanistic rationale for the compromised performance of NPBA at high temperatures.

Solid-state NMR (SSNMR) has been proved to be an indispensible technique to investigate the microstructural properties and dynamic behaviors of insoluble and non-crystalized biological systems, carbohydrate complexes and advanced functional materials [16,17,18,19,20,21,22,23,24,25,26,27,28,29,30,31,32,33,34]. Specifically, an array of techniques, including DIPSHIFT [35,36], 2D WISE [37], SUPER [38], ^2^H-NMR [39], REDOR [40,41] and relaxation parameters measurements [42], have been well established to investigate the rigidity or mobility of a variety of functional (bio-)polymers. In recent years, lots of efforts have been made towards better understanding the structure-property relationship of different kinds of polymeric species at the atomic or molecular level using SSNMR. For example, Kameda and Tsukada [43] utilized versatile SSNMR techniques to investigate the physical properties and chemical structure of silk fibers grafted with methacrylamide. The chain conformations and dynamics of crystalline polymers forming inclusion compounds have been successfully elucidated by SSNMR [44]. The domain structure and mobility of poly(propylmethacryl-heptaisobutyl-pss)-co-styrene nanocomposites with different polyhedral oligomeric silsesquioxane (POSS) contents were investigated by SSNMR techniques including 2D WISE [37] and spin diffusion [45]. In addition, structures and thermal properties of the multiple ordered phases in ethylene-octene and ethylene-butene copolymers have been studied using the measurements of ^13^C chemical shift tensors and NMR relaxation times [46].

In this work, we utilize SSNMR to study the molecular dynamics of NPBA, an efficient bonding polymer used in propellants and firstly synthesized by Kim [11], as a function of various temperatures. Meanwhile, the structural and dynamic perturbations of NPBA upon aging at high temperatures up to 423 K are investigated. In addition, we examine the structural change upon the treatment of acetone, which is frequently used as a dispersant for NPBA in the synthesis and propellant processing. 

## 2. Results and Discussion

### 2.1. Characterization of NPBA by 1D ^13^C CP/MAS NMR Spectroscopy

**Figure 1:**
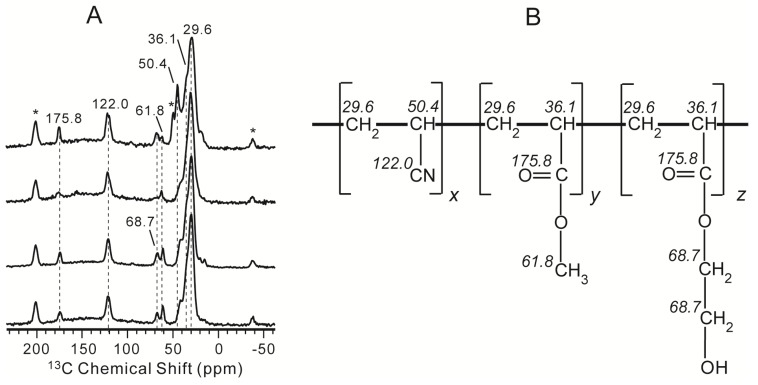
(**A**) ^13^C CP/MAS NMR spectra of the NPBA acquired at room temperature and under 6 kHz MAS. Asterisks (*) represent spinning sidebands; (**B**) Schematic molecular structure of the NPBA, with assigned chemical shifts for different carbon sites. The broad base line at approximately 150 ppm arises from the probe background. The x, y, z values in NPBA are ca. 1.0, 0.2, and 0.2, respectively.

We firstly obtained the 1D ^13^C CP/MAS spectrum of NPBA to evaluate the sample homogeneity and assign the resonances of various carbon groups. As shown in Figure 1A, the 1D ^13^C spectrum shows characteristic features, including high intensity of CH and CH_2_ peaks at 20–45 ppm and downfield resonances of CN and CO groups, respectively, at 122.0 ppm and 175.8 ppm, in good agreement with the structural motif of NPBA. The simple spectral features also suggest that the sample is very unlikely to show large inhomogeneity. Based on the characteristic values and studies of polymers containing similar functional segments [47,48], all peaks are assigned. The peaks at 29.6 and 36.1 ppm were respectively attributed to the methine and methylene groups present in the main chain. The peak at 122.0 ppm was assigned to the only CN group [49]. The chemical shift at 50.4 ppm could be tentatively assigned to the CH group directly bonded to CN. The signal at 175.8 ppm was from C=O of the ester groups [50]. The peak at 68.7 ppm was ascribed to methylenes in the side chain. The signal at 61.8 ppm was assigned to methyl groups in the methacrylate side chain segment. All assigned NMR chemical shifts are indicated in the schematic structure of NPBA in Figure 1B.

### 2.2. ^1^H-NMR Spectra of NPBA Showing Distinct Dynamics Above and Below the PhaseTransition Temperature

**Figure 2:**
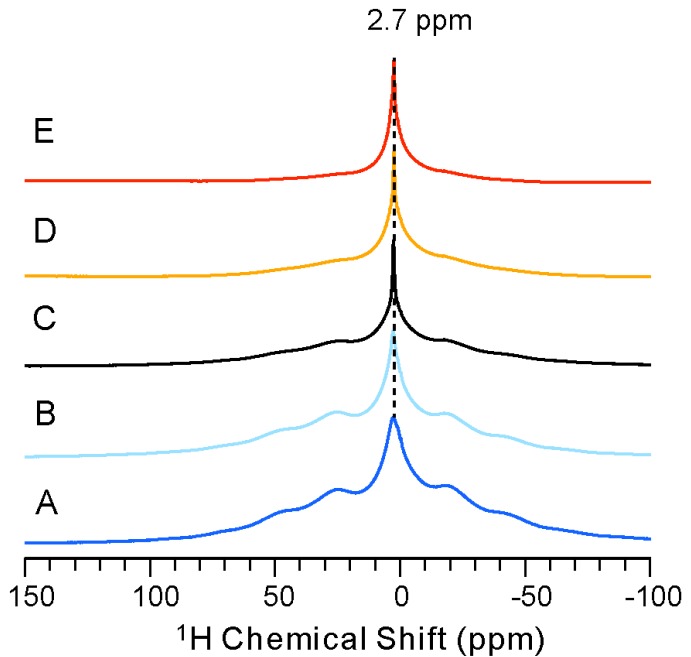
^1^H MAS NMR spectra of NPBA at five different temperatures: (**A**) 298 K; (**B**) 318 K; (**C**) 333 K; (**D**) 353 K and (**E**) 363 K.

In order to investigate the structural perturbation upon heating, we conducted a variable temperature (VT) study of NPBA. Figure 2 shows its ^1^H spectra at five different temperatures of 298 K, 318 K, 333 K, 353 K and 363 K. As the shared feature, all spectra show a major peak centered at 2.7 ppm along with the spinning sidebands and are largely broadened due to the strong ^1^H homonuclear dipole interaction. The central peak clearly experiences line narrowing when the temperature increases from 298 K to 353 K, suggesting faster motions at higher temperatures. A significantly narrowed peak is obtained at 353 K (Figure 2E). In addition, the broader shoulder decreases with increasing temperature and nearly disappears at 353 K. As measured by differential scanning calorimeter (DSC), NPBA shows a glass transition temperature, T_g_, at approximately 329 K. On one hand, the distinct spectra feature between A–B and C–E suggest the faster motion above the T_g_. On the other hand, the broad line-width of the central peak below T_g_ indicates stronger ^1^H-^1^H dipole interactions, suggesting the higher rigidity of NPBA. Since the ^1^H spectra show the lack of resolution to provide site-specific dynamics, we then acquired the ^13^C-detected CP/MAS spectra of NPBA at various temperatures.

### 2.3. Site-Specific Dynamics of NPBA Obtained from ^13^C VT CP/MAS Spectra

**Figure 3:**
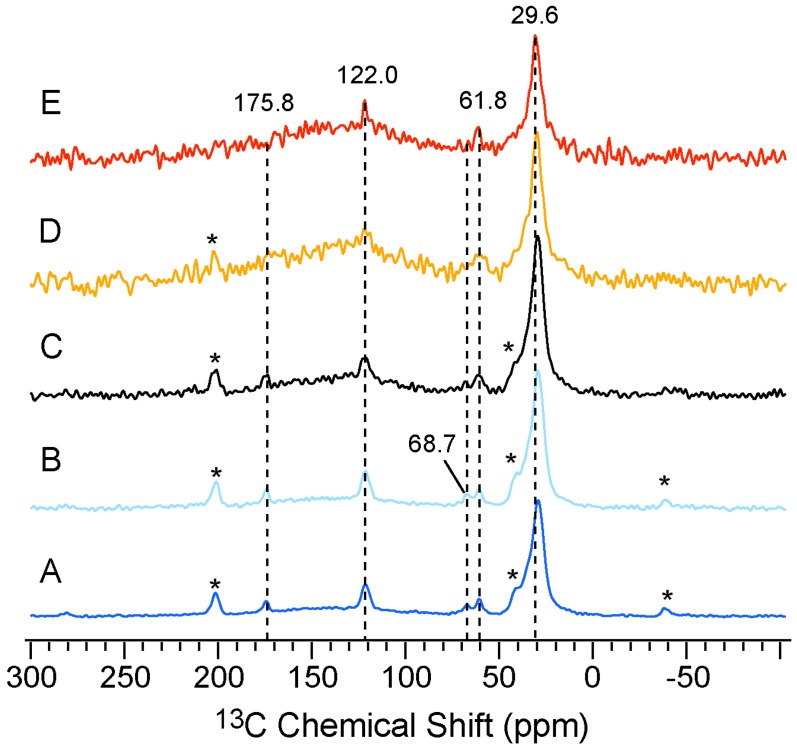
^13^C CP/MAS NMR spectra of NPBA at five different temperatures: (**A**) 298 K; (**B**) 318 K; (**C**) 333 K; (**D**) 353 K and (**E**) 363 K.

To further study the temperature-dependent dynamic change and probe the structural perturbation at high temperatures, ^13^C-detected VT experiments are carried out. As shown in Figure 3, the chemical shifts of different carbon sites remain unchanged upon heating, suggesting a stable structure of NPBA below the glass transition. Interestingly, peaks at 68.7 ppm, 122.0 ppm and 175.8 ppm (assigned to side-chain CH_2_, CN and CO, respectively) are well resolved in Figure 3A,B, but become very broad in Figure 3D,E. These observations indicate that side chains adopt different motion at temperatures higher than T_g_, e.g., likely the intermediate motion interferes with the ^1^H decoupling and thus gives broad lines [31,51]. Alternatively, the side chains could experience conformational disorder at above the T_g_, resulting in inhomogeneous line broadening. In contrast, the ^13^C peak of the main-chain CH_2_ at 29.6 ppm shows a consistent line width and intensity in spectra acquired above and below the T_g_, suggesting a relatively rigid polymer backbone in the studied range of temperatures. To quantitatively characterize the dynamic behavior of NPBA, we then measured the ^13^C chemical shift anisotropy (CSA) and dipolar coupling of the C-H bond. The comparisons between the motion-averaged strength of these spin interactions and the value at a rigid limit can provide better understanding of the backbone and side chain motions.

### 2.4. Measurement of the Motion-Averaged ^13^C CSA

**Figure 4:**
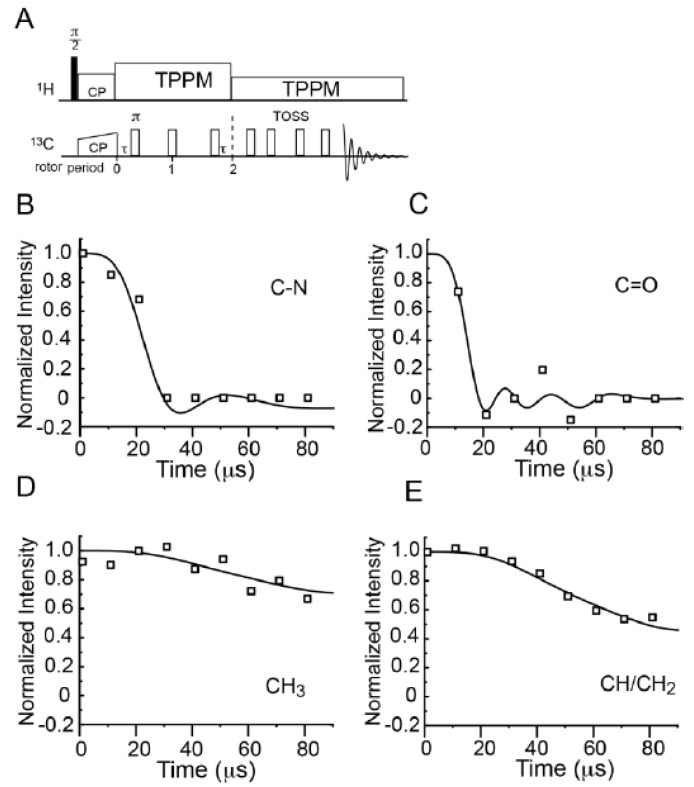
The pulse sequence (**A**) and numerical fittings (**B**–**E**) of CSA measurements of four different carbon sites in NPBA: (**A**) CN; (**B**) CO; (**C**) CH_3_ and (**D**) CH/CH_2_.

The ^13^C CSA of different groups are measured using a five-pulse scheme developed by Mao *et al.* [52] to examine the dynamics of NPBA at room temperature (see the pulse sequence in Figure 4A). The experimental data and numerical fitting for four different groups including CN, CO, CH_3_ and CH/CH_2_ were shown in Figure 4B–E. The CSA simulation results were summarized in Table 1. The CN and CO groups have relatively large CSA values at 75 and 150 ppm, compared with the same groups in other rigid systems [53,54]. This confirms the rigidity of the side chains of NPBA at room temperature. The ^13^C CSA values of CH/CH_2_ are intrinsically small and thus not suitable to sense the motion of the polymer backbone. To investigate the motion of the main chain at room temperature and the dynamic properties of NPBA at temperatures higher than T_g_, we measured the dipolar coupling of C-H bonds at various temperatures.

**Table 1:** Simulated CSA values of different carbon groups from Figure 4. The unit of CSA values is in ppm. The errors were deduced in the SIMPSON simulations.

Groups	C-N	C=O	CH_3_	CH/CH_2_
CSA	75 ± 8	150 ± 20	11 ± 2	16 ± 2

### 2.5. Rigidity of NPBA Evaluated by the Motion-Averaged ^13^C-^1^H Dipolar Coupling

**Figure 5:**
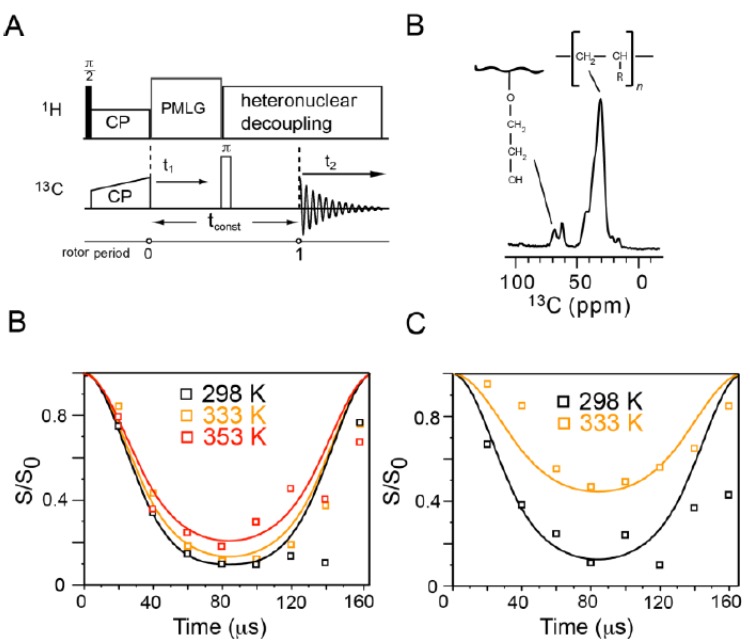
Measurement of motion-averaged ^13^C-^1^H dipolar couplings of NPBA. (**A**) The NMR pulse sequence of the DIPSHIFT experiment; (**B**) Representative 1D projection of the DIPSHIFT spectrum, showing the resonances of backbone and side-chain CH_2_; (**C**) The curve fitting of backbone CH_2_ data measured at 298 K, 333 K and 353 K; (**D**) Fitted side-chain CH_2_ data measured at 298 K and 333 K. Time on x-axis refers to the evolution time in t1 period.

The C-H dipolar coupling is measured using the DIPSHIFT experiment (Figure 5A), which allows the evolution of magnetization under dipolar interaction in one rotor period. The dipolar order parameter is the ratio of the measured dipolar coupling to the rigid limit, indicating the amplitude of motion (as represented in θ). For example, an order parameter of 1.0 corresponds to the rigid limit while a small order parameter near 0 indicates large-amplitude, e.g., isotropic motion. Main chain and side chain CH_2_ peaks at 29.6 ppm and 68.7 ppm, respectively, are well resolved and used to read out the intensity that is modulated by C-H dipolar interaction in the DIPSHIFT experiment, as shown in Figure 5B–D. The fact that the ^13^C intensity does not fully recover at the end of the rotor period and shows minor decay is due to the apparent T_2_ decay at a longer time (Figure 5C). The measured ^13^C-^1^H dipolar coupling (ω_CH_) and derived order parameters (S_CH_) are tabulated in Table 2. At 298 K, the main-chain and side-chain CH_2_ have dipolar couplings of 24.3 kHz and 21.7 kHz, respectively, giving order parameters close to 1.0. These results indicate the high rigidity of NPBA molecules at room temperature, which agrees well with the finding of the ^13^C CSA measurement. When it comes to 333 K, the backbone is still relative rigid (S_CH_ = 0.96 and a small motional angle at 9.4 degrees), while the side chain becomes mobile (S_CH_ = 0.61 and a relatively large motional angle at 30.7 degree). At an even higher temperature of 353 K, the main chain starts becoming a little bit flexible (S_CH_ = 0.84). Taken together, the side chain shows large mobility at above the glass transition temperature, whilst the main chain remains relatively rigid in the phase change from 298 K to 353 K. These results are consistent with the ^13^C VT CP/MAS NMR observations in Section 2.3.

**Table 2:** Dipolar order parameters (S_CH_) of backbone and side-chain CH_2_ groups measured by the 2D ^13^C-^1^H DIPSHIFT experiment. The angle of motional amplitudes (θ) is in degree.

CH_2_ Groups	Main Chain			Side Chain	
	298 K	333 K	353 K	298 K	333 K
ω_CH_ (kHz)	24.3	20.8	19.1	21.7	13.9
S_CH_ *	1.07	0.92	0.84	0.96	0.61
Motional amplitude (θ)	0	13.4	19.1	9.4	30.7

* The dipolar order parameter, S_CH_, is the ratio of measured ω_CH_ to the rigid limit of 22.7 kHz, indicating the motional amplitude between the motional axis and C-H bond.

### 2.6. Structural Perturbation of NPBA upon Aging and Acetone Soaking

**Figure 6:**
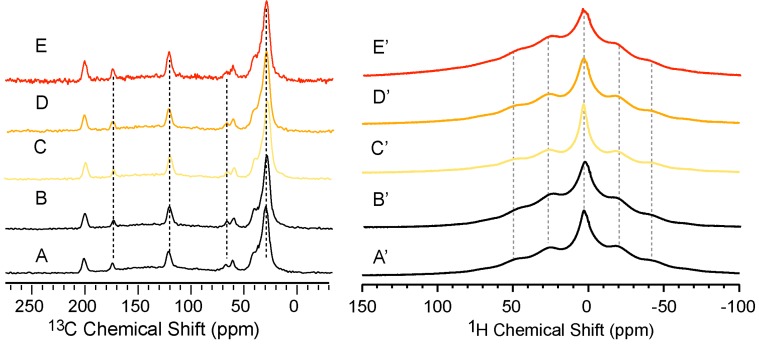
^13^C CP/MAS (left panel) and ^1^H (right panel) spectra of NPBA after acetone treatment or aging. (**A** and **A'**) NPBA without further treatments as the control spectrum. (**B** and **B'**) Acetone-treated NPBA. (**C**–**E** and **C'**–**E'**) NPBA aged for 16 h at different temperatures: 333K (**D** and **D'**), 353 K (**E** and **E'**) and 423 K (**F** and **F'**).

To investigate the effect of acetone treatment and aging on the structural properties of NPBA, four different samples are prepared and used for ^13^C CP/MAS and ^1^H measurements shown in Figure 6. Surprisingly, no apparent spectral difference is observed by comparing the control (A), acetone-treated (B) and aged (C–E) samples. The same consistency is observed for ^1^H spectra on the right panel of Figure 6. Both ^13^C CP/MAS and ^1^H spectra suggest that the microstructure of NPBA remains stable after various aging and acetone treatments. 

## 3. Experimental

### 3.1. Sample Preparation

Polymer samples were synthesized according to a previous literature report [11]. In brief, acrylonitrile, methacrylate and hydroxyethylacrylate were mixed at a desired molar ratio and polymerized at 333 K. Azodiisobutyronitrile and mercaptoethanol serve as initiator and molecular weight adaptor, respectively. The polymerization reaction lasted for ca. 6 h with stirring and refluxing in acetone. After filtration, the solid phase was dehydrated at 323 K for 15 h, giving the NPBA product as a white power. The average block length and monomer distribution in the synthesized copolymer BPBA is determined to be around 50. The momomer should be randomly distributed in NPBA in amorphous forms. The glass transition temperature of the synthesized product is approximately 329 K as determined by differential scanning calorimeter (DSC). In order to study the structural perturbation of NPBA at different temperatures, three samples were made via conditioning in aging ovens for 12 h at temperatures of 333 K, 353 K, and 423 K. In addition, 0.5 g NPBA was immersed in 20 mL acetone for 1 h and subsequently dried in the air, for the purpose of determining the stability of NPBA after acetone treatment. 

### 3.2. Solid State NMR Experiment

All solid-state NMR experiments were carried out on a Varian Infinityplus-300 spectrometer equipped with a 4 mm double-resonance MAS probe. The operating Larmor frequencies are 299.8 and 75.4 MHz respectively for ^1^H and ^13^C nuclei on a 300 MHz spectrometer. Typically, rf (radio frequency) field strengths were 35–50 kHz for ^13^C and 42–83 kHz for the ^1^H channel. ^1^H and ^13^C chemical shifts were externally referenced to the adamantine. The ^13^C cross-polarization magic angle spinning (CP/MAS) NMR experiments were conducted using a contact time of 1.5 ms and under 6 kHz MAS. The ^1^H MAS NMR spectra were obtained with single pulse at 6.5 kHz MAS. 

The ^13^C chemical shift anisotropy (CSA) of different samples was determined by using the five-pulse CSA-recoupling sequence, a robust ^13^C selecting pulse scheme in use of carbon bonding symmetry as introduced by Schmidt-Rohr *et al.* [52]. In the ^13^C CSA measurement, there are three π pulses in two rotor periods, where the second π pulse is fixed in the middle of one rotor period, whereas the delay between the first π pulse and initial as well as the third π pulse to the end were varied simultaneously as manifested in the Figure 4. A π pulse length of 10 μs was utilized during the ^13^C or ^15^N CSA measurements. The measurements were conducted under 6 kHz MAS. The CSA anisotropy parameter δ for each ^13^C site were simulated by using the SIMPSON software package [55]. In the ^13^C CSA measurement, there are three π pulses in two rotor periods, where the positions of initial and last π pulses vary upon different τ, and the second π pulse is fixed in the middle of one rotor period. The pulse program is simulated using SIPMSON, which indicates that the fitting remarktably depends on the ^13^C CSA value and is insensitive to Euler angles or asymmetry parameters. The motional averaged ^13^C–^1^H dipolar couplings were measured using the Dipolar-chemical-shift (DIPSHIFT) experiment under 6 kHz MAS [35]. The pulse block of PMLG [56] was used to achieve ^1^H homonuclear dipolar decoupling during the rotor period. The TPPM scheme was used to achieve 80 kHz ^13^C-^1^H decoupling. The time domain data were fit to give the apparent dipole coupling values and then were divided by PMLG scaling factor to derive the true coupling strength (ω_CH_). The scaling factor of the PMLG decoupling with transverse filed strength of 80 kHz was experimental calibrated to be 0.54.

## 4. Conclusions

The performance of NPBA agents relies remarkably on their molecular microstructures and interactions to the bonded matrix [11,12]. It has been proposed that the functional groups in the NPBA side chain could readily react with the binder. Additionally, the presence of NPBA forms a high modulus layer around the oxidant fillers, which eliminate the direct interaction upon dewetting between binder matrix and oxidant fillers. This architectural arrangement required for a functional propellant could be corrupted under severe conditions, e.g., at high temperatures, due to debonding between NPBA and the binder matrix. We utilized solid-state NMR to characterize the structure and dynamics of a NPBA polymer at different temperatures and after treatment with acetone and aging. The ^13^C VT CP/MAS spectra and NMR parameters of ^13^C CSA and S_CH_ show that main chain of the studied polymer remains relatively rigid at temperatures up to 333 K. Interestingly, the side chains are rigid at room temperature but become mobile at temperatures above T_g_. The identified rigidity of NPBA molecule below the T_g_ presumably provides a structural basis for the stable binding between its side chains and binder matrix. At temperatures higher than the Tg, e.g., 333 K, such binding becomes weak due to the enhanced mobility of the side chain, as our dynamic results indicated. In addition, the flexible side chains at high temperatures result in the reduction of the thickness of the modulus layer around the oxidant. The temperature-induced dynamic change of NPBA results in its compromised performance at high temperatures. This also suggests that the development of bonding agents with high T_g_ could significantly improve the thermal stability, which is crucial to maintaining the interaction between bonding agent and binder matrix. Moreover, even though the NPBA has been aged at temperatures above T_g_, ^1^H and ^13^C-NMR spectra remains unchanged, indicating that the microstructure of NPBA was considerably stable and shows no deformation at high temperatures up to 423 K. NPBA also shows no spectroscopic difference before and after soaking with acetone, suggesting the use of this organic solvent as dispersant causes no structural perturbation to the NPBA polymer. These findings provide a new insight into the storage and production processing of NEPE propellants. 

Most of the previous studies on propellant bonding have mainly focused on engineering properties and scarcely emphasized the investigation of the molecular microstructure and dynamics. Here we utilize solid-state NMR spectroscopy to characterize the structure and dynamics of a NPBA polymer at various temperatures and with different aging and acetone treatments. Our results indicated that both the main-chain and side-chain in the microstructure of NPBA remained relatively rigid below T_g_, whilst the molecular side chains become flexible at higher temperatures. These structural and dynamic findings rationalize the stability of NPBA serving as a bonding agent at temperatures below T_g_ and its compromised performance at higher temperatures. Our study has shown that solid-state NMR is a robust and efficient analytical technique to characterize the structure and dynamics of NPBA polymers. Moreover, the findings provide the insight of improving the mechanical property of NEPE propellants at high temperature by increasing the T_g_ of the bonding agent. 
